# Supplementary material for: A high-content screen of FDA approved drugs to enhance CAR T cell function: ingenol-3-angelate improves B7-H3-CAR T cell activity by upregulating B7-H3 on the target cell surface via PKCα activation
Source: J Exp Clin Cancer Res. 2024 Apr 1;43:97. doi: 10.1186/s13046-024-03022-x (PMC10985962; doi:10.1186/s13046-024-03022-x)
Supplement: Supplementary file 1 — Supplementary Material 1. [file 13046_2024_3022_MOESM1_ESM.docx]

Supplementary Materials for

**A high-content screen of FDA approved drugs to enhance CAR T cell function: ingenol-3-angelate improves B7-H3-CAR T cell activity by upregulating B7-H3 on the target cell surface via PKCα activation**

Ha Won Lee,^1^ Carla O’Reilly,^2^ Alex N. Beckett,^2,3^ Duane G. Currier,^1^ Taosheng Chen,^1^ Christopher DeRenzo,^2^

^1^Department Chemical Biology and Therapeutics, St. Jude Children’s Research Hospital, Memphis, TN, USA, 38105.

^2^Department of Bone Marrow Transplantation and Cellular Therapy, St. Jude Children’s Research Hospital, Memphis, TN, USA, 38105.

^3^Graduate School of Biomedical Sciences, St. Jude Children’s Research Hospital, Memphis, TN, USA, 38105.

Correspondence to: [chris.derenzo@stjude.org](mailto:hawon.lee@stjude.org)

**This file includes:**

Figures. S1-S15

Table S1

**Figure S1.**

**
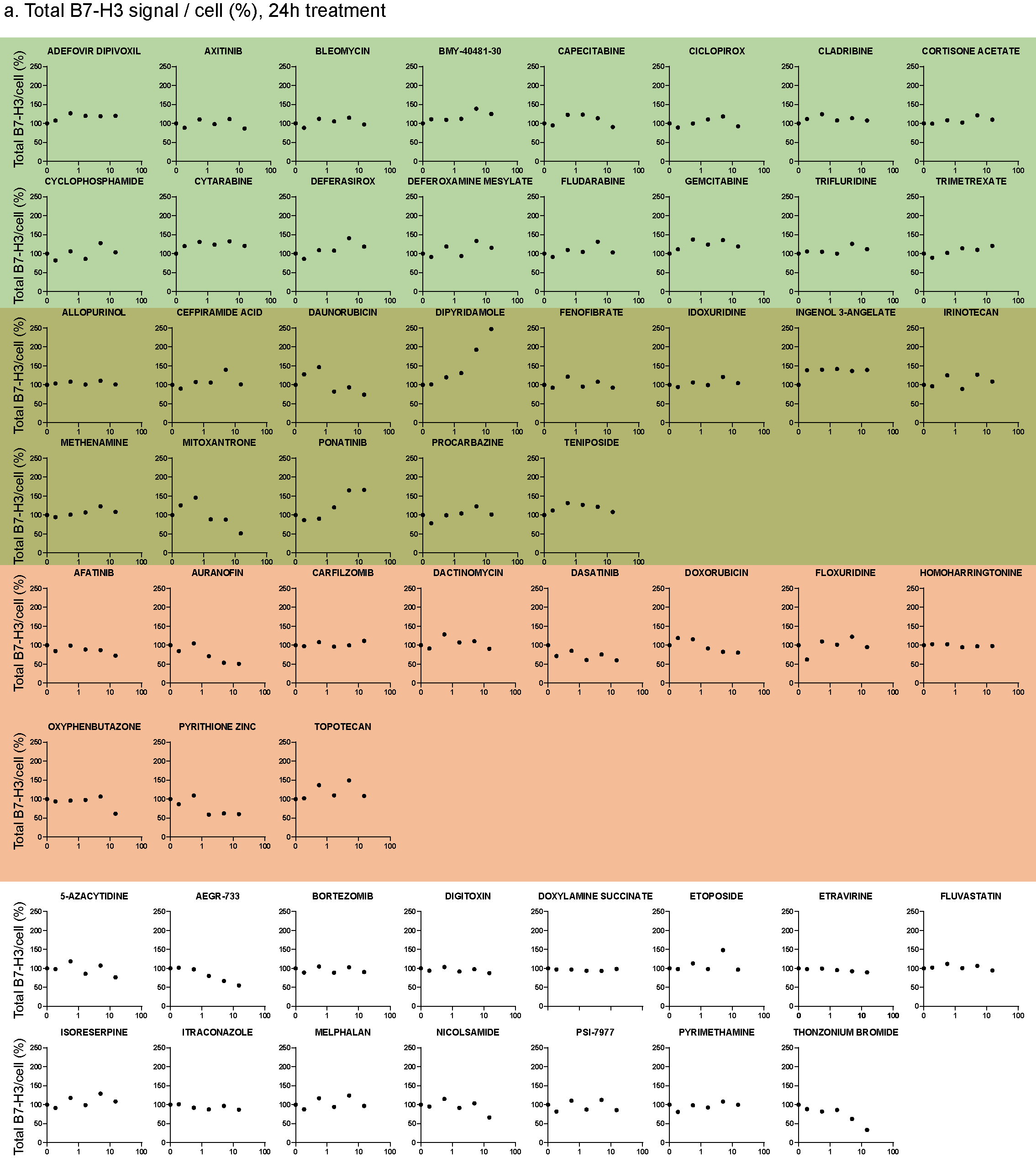
**

**Figure S1.**

**
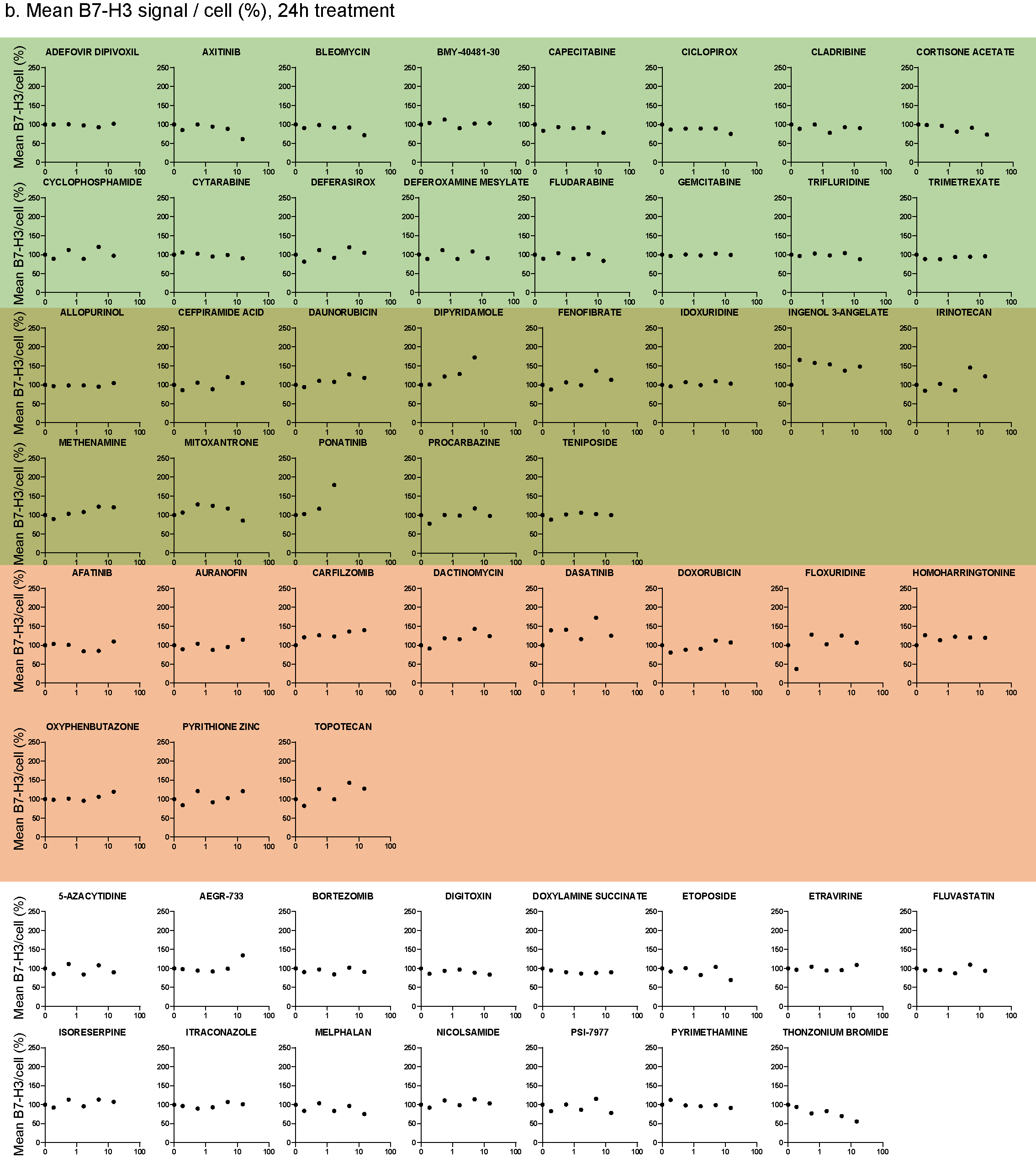
**

**Figure S1.**

**
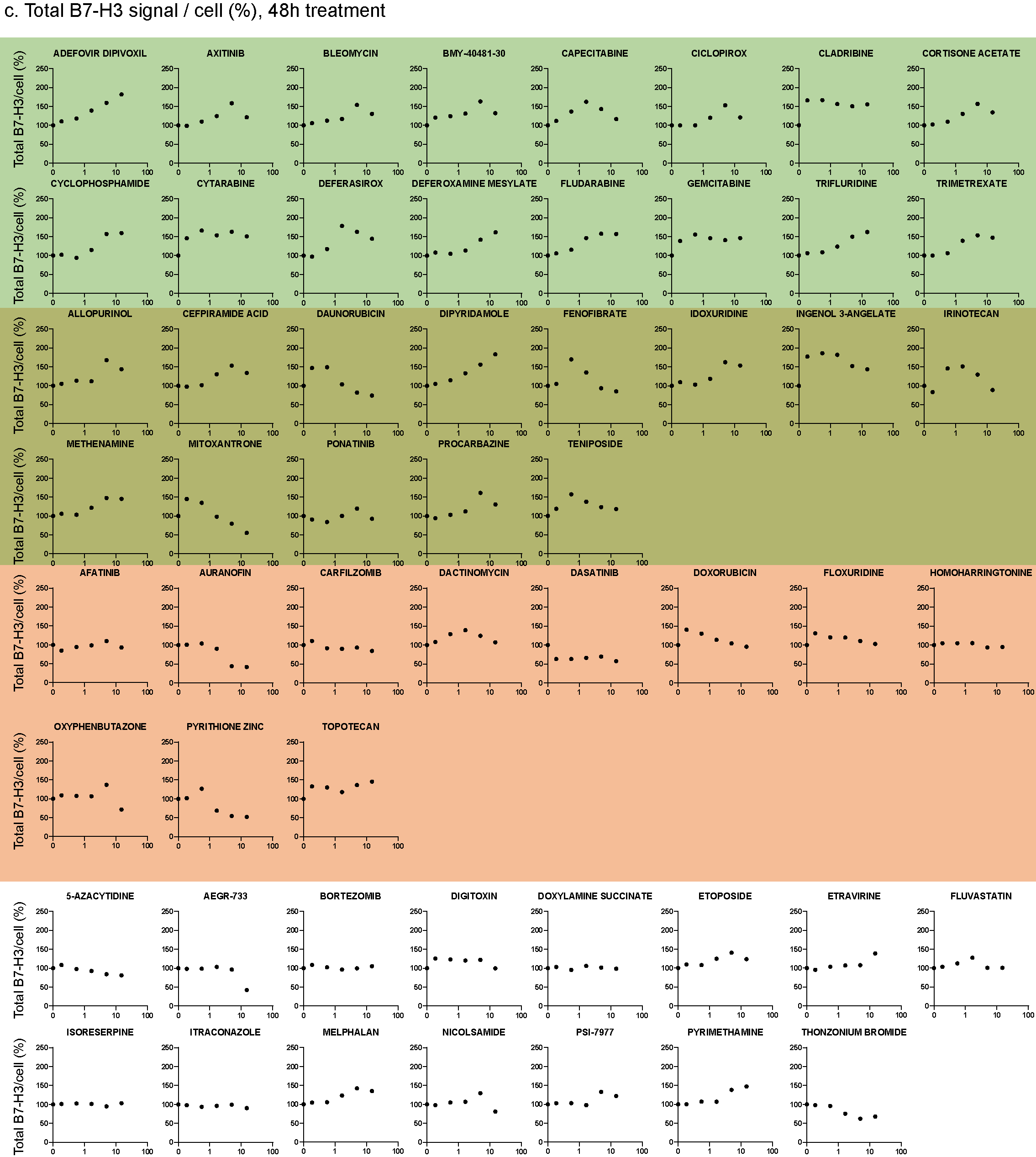
**

**Figure S1.**

**
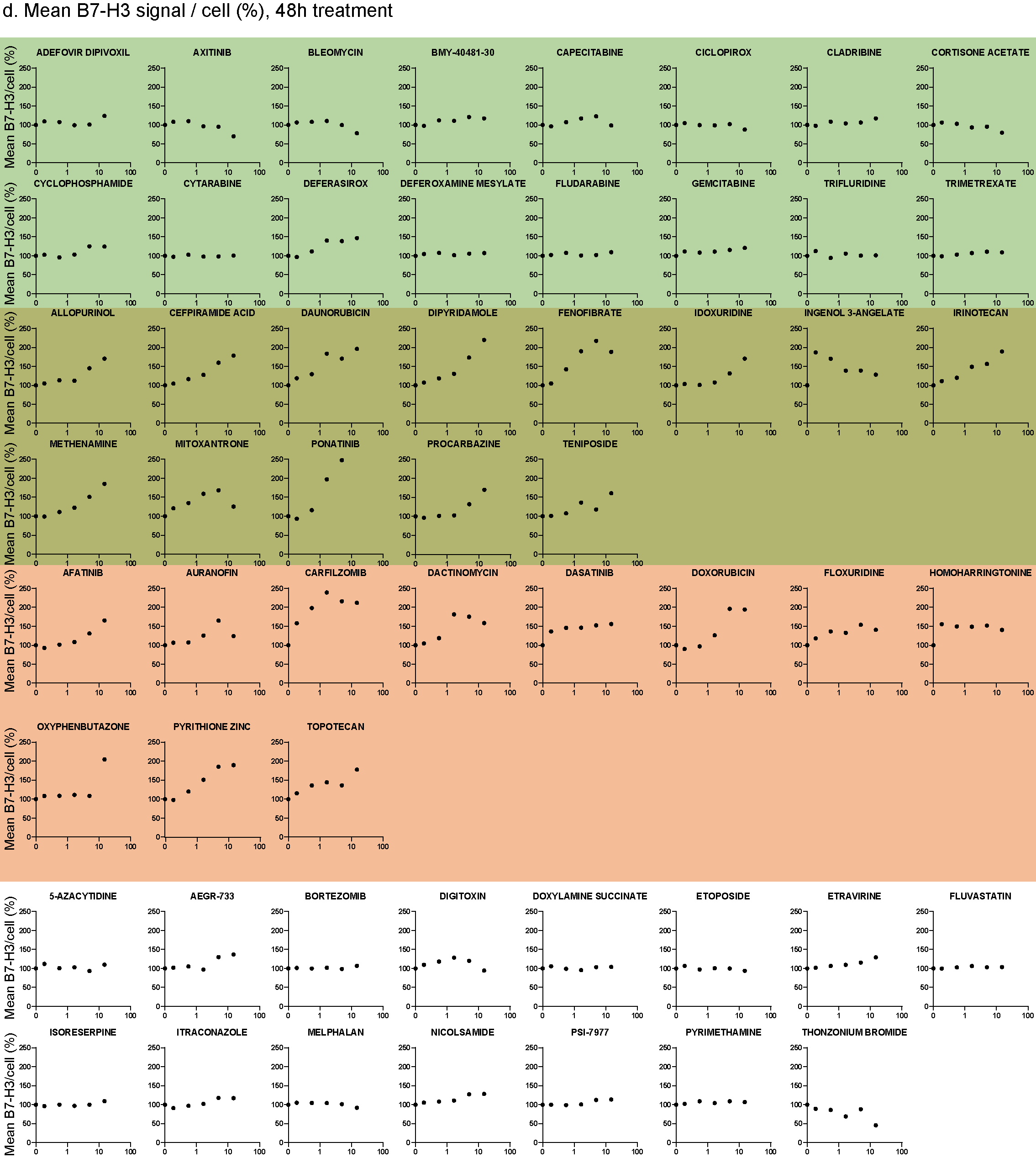
**

**Figure S1. Five-point dose-response assay for compounds identified to increase B7-H3 in LM7 cells**. Five doses of 55 compounds were treated on LM7 cells and B7-H3 was quantified using the immunofluorescence assay. **a** Total B7-H3/cell after 24 hrs. **b** Mean B7-H3/cell after 24 hrs. **c** Total B7-H3/cell after 48 hrs. **d** Mean B7-H3/cell after 48 hrs. Forty compounds increased the total and/or mean B7-H3/cell greater than 50% at one or more doses at 24 or 48 hrs. Background color: green, increased total B7-H3 only; dark green, increased total and mean B7-H3; red, increased mean B7-H3 only; white; no increase in total or mean B7-H3 greater than 50%.

**Figure S2.**

**
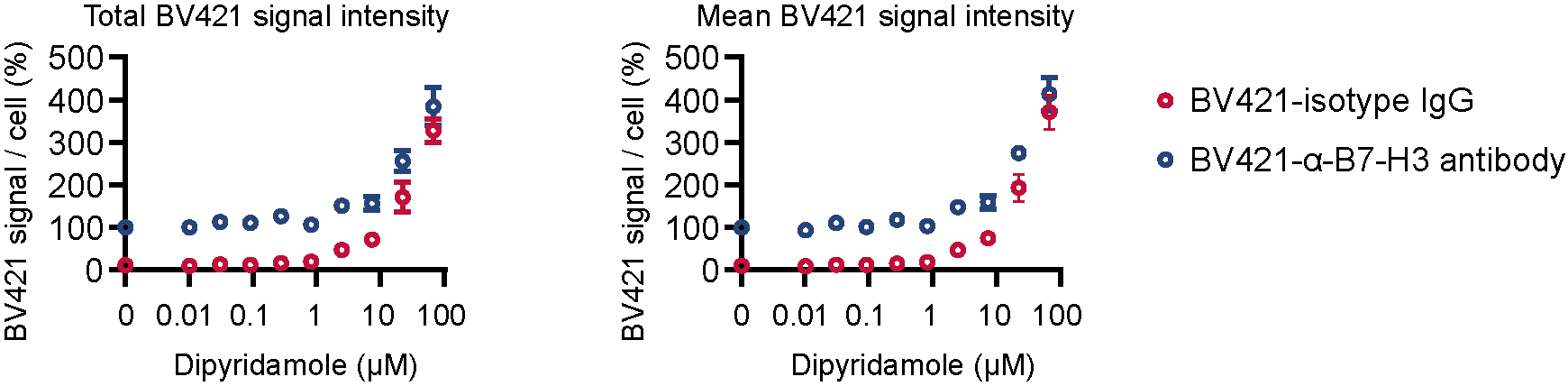
**

**Figure S2. Dipyridamole non-specifically increases the fluorescent signal in LM7 cells.** LM7 cells were treated with 10 doses of dipyridamole for 48 hours and the BV421 signal intensity was measured by the immunofluorescence assay using the BV421-α-B7-H3 antibody or BV421-isotype IgG control (N=4). The BV421 signal per cell was normalized by averages of DMSO treated wells that were stained with BV421-α-B7-H3 antibody. Data represents mean ± SD.

**Figure S3.**

**
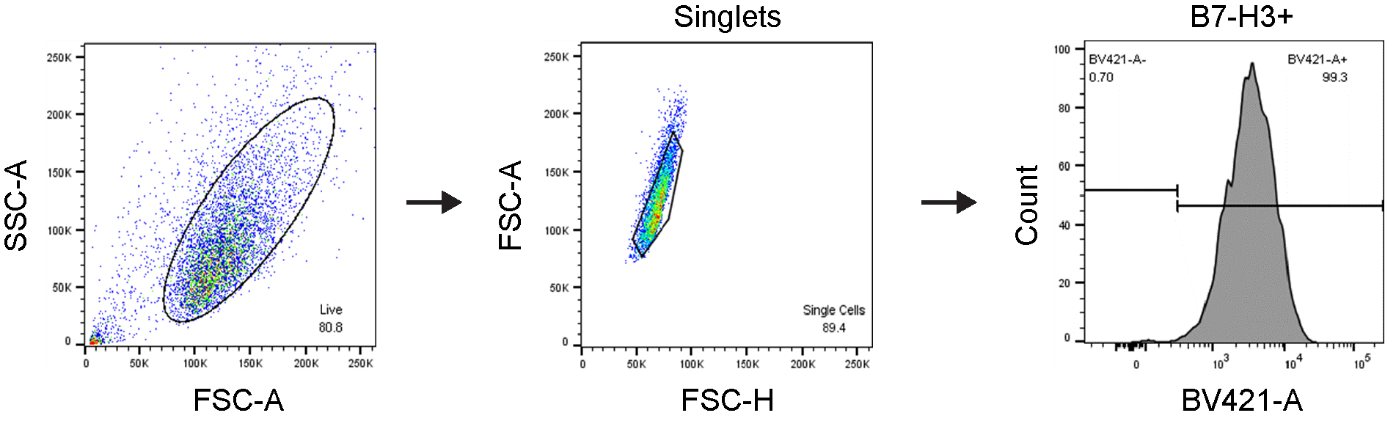
**

**Figure S3. Flow cytometry gating strategy for Main Figure 2b.** Representative flow cytometry plots of LM7 cells treated with 0.1uM ingenol-3-angelate (I3A) for 48 hours are shown to demonstrate the gating strategy used for experiments depicted in Main Figure 2b.

**Figure S4.**


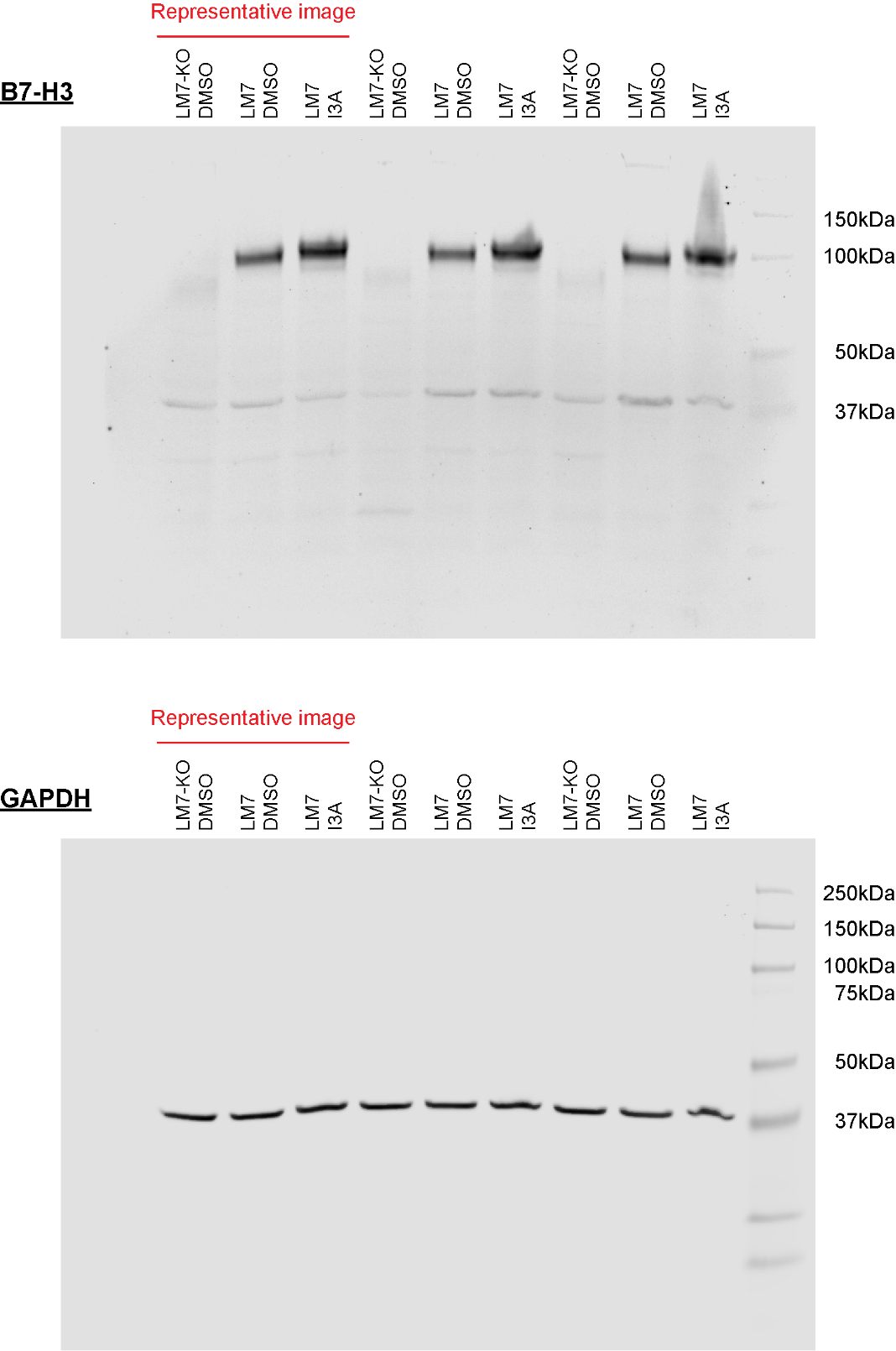


**Figure S4. Uncropped western blot images of representative images in Figure 2d.** Three biological triplicates are blotted on a single membrane. The representative blots shown in Figure 2d are highlighted by red lines over the lanes. Molecular weight markers are shown in the rightmost lane, and their molecular weights are labeled on the right.

**Figure S5.**


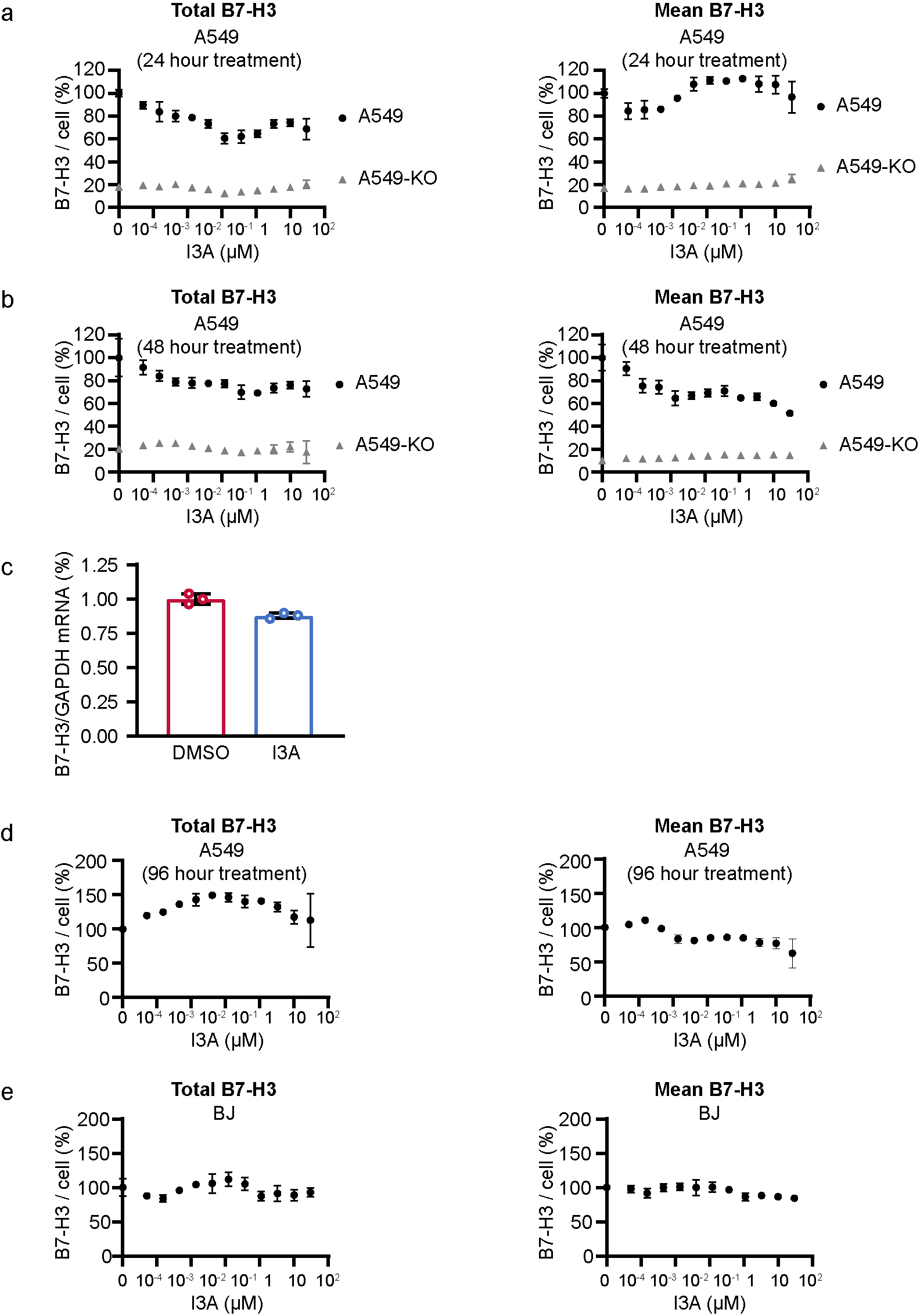


**Figure S5. Ingenol-3-angelate (I3A) does not increase B7-H3 in lung adenocarcinoma or normal human fibroblast cell lines.** **a,b** B7-H3 expression per cell in A549 (lung adenocarcinoma) or B7-H3 knockout A549 (A549-KO) cells after treatment with 12 doses of I3A for **a** 24 or **b** 48 hours (N=3). **c** B7-H3 mRNA expression in A549 cells treated with 0.5µM I3A for 48 hours. The B7-H3 mRNA level was normalized by the GAPDH mRNA level (N=3). **d** B7-H3 expression per cell in A549 cells after treatment with 12 doses of I3A for 96 hours (N=4). **e** B7-H3 expression per cell in BJ (human fibroblast) cells after treatment with 12 doses of I3A for 48 hours (N=4). Data represents mean ± SD (a-e).

**Figure S6.**


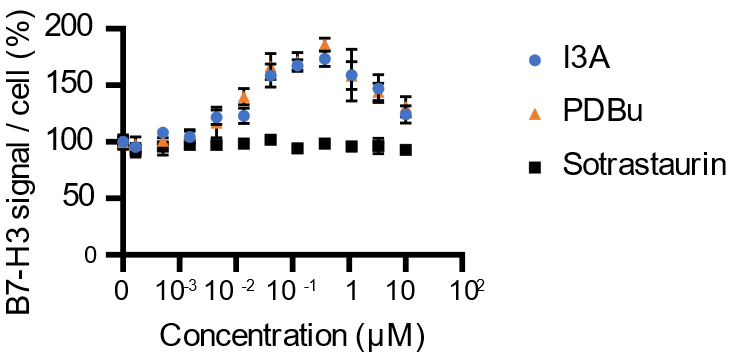


**Figure S6. The PKC antagonist sotrastaurin does not change B7-H3 protein expression in LM7 cells.** LM7 cells were treated with 12 doses of I3A, PDBu, or sotrastaurin for 48 hours and B7-H3 protein expression determined by the immunofluorescence assay (N=4). The total B7-H3 signal per cell was normalized by averages of DMSO-treated wells. Data represents mean ± SD.

**Figure S7.**


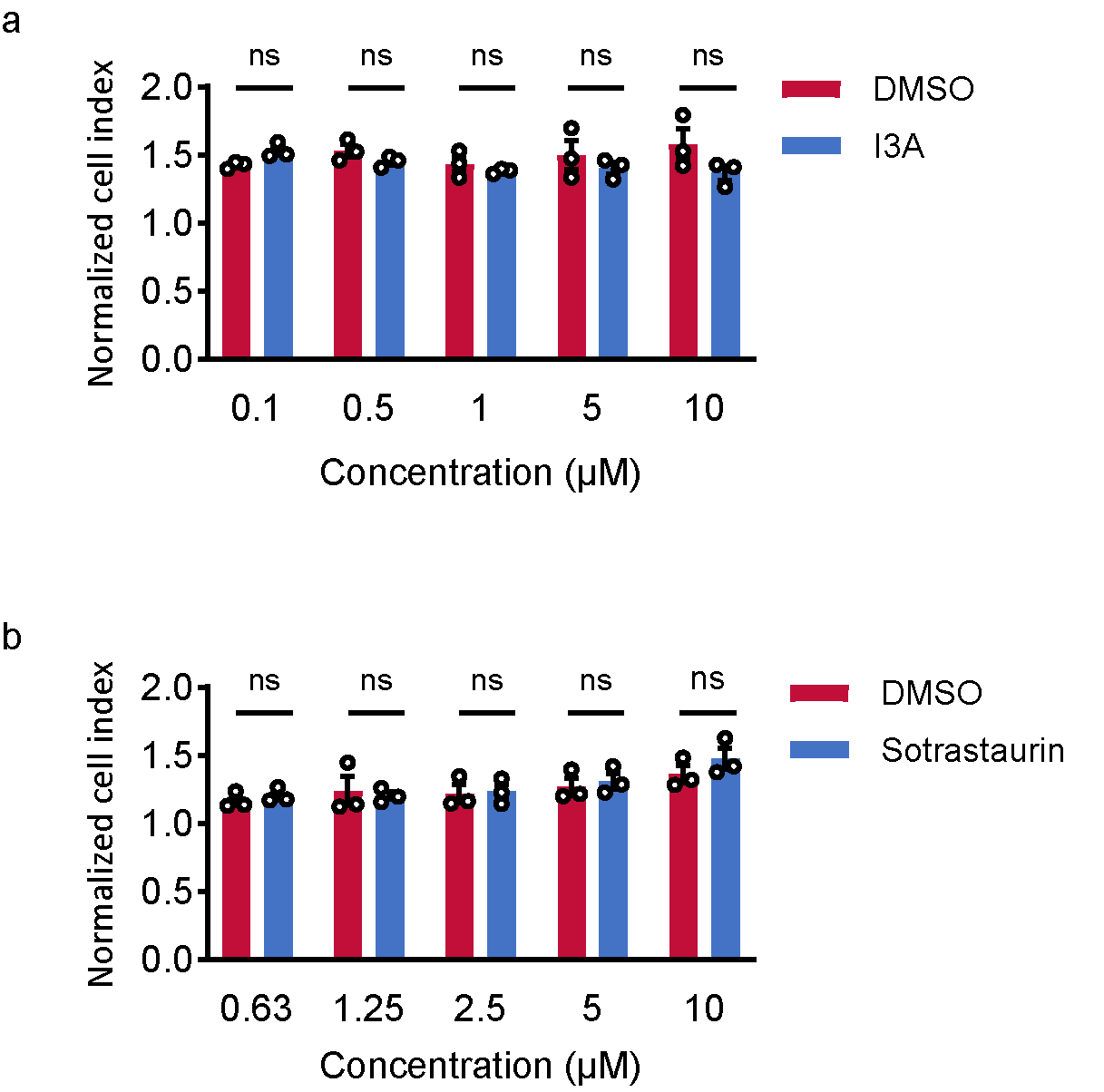


**Figure S7. I3A and sotrastaurin do not inhibit LM7 cell growth.** LM7 osteosarcoma cells were treated with DMSO, **a** ingenol-3-angelate (I3A; N=3 technical replicates) or **b** sotrastaurin (N=3 technical replicates) for 48 hours at indicated concentrations. Cell growth/inhibition was quantified using an impedance-based assay (xCELLigence). Data represents mean ± SEM (a, b). ns, non-significant by multiple paired t-tests (a, b).

**Figure S8.**


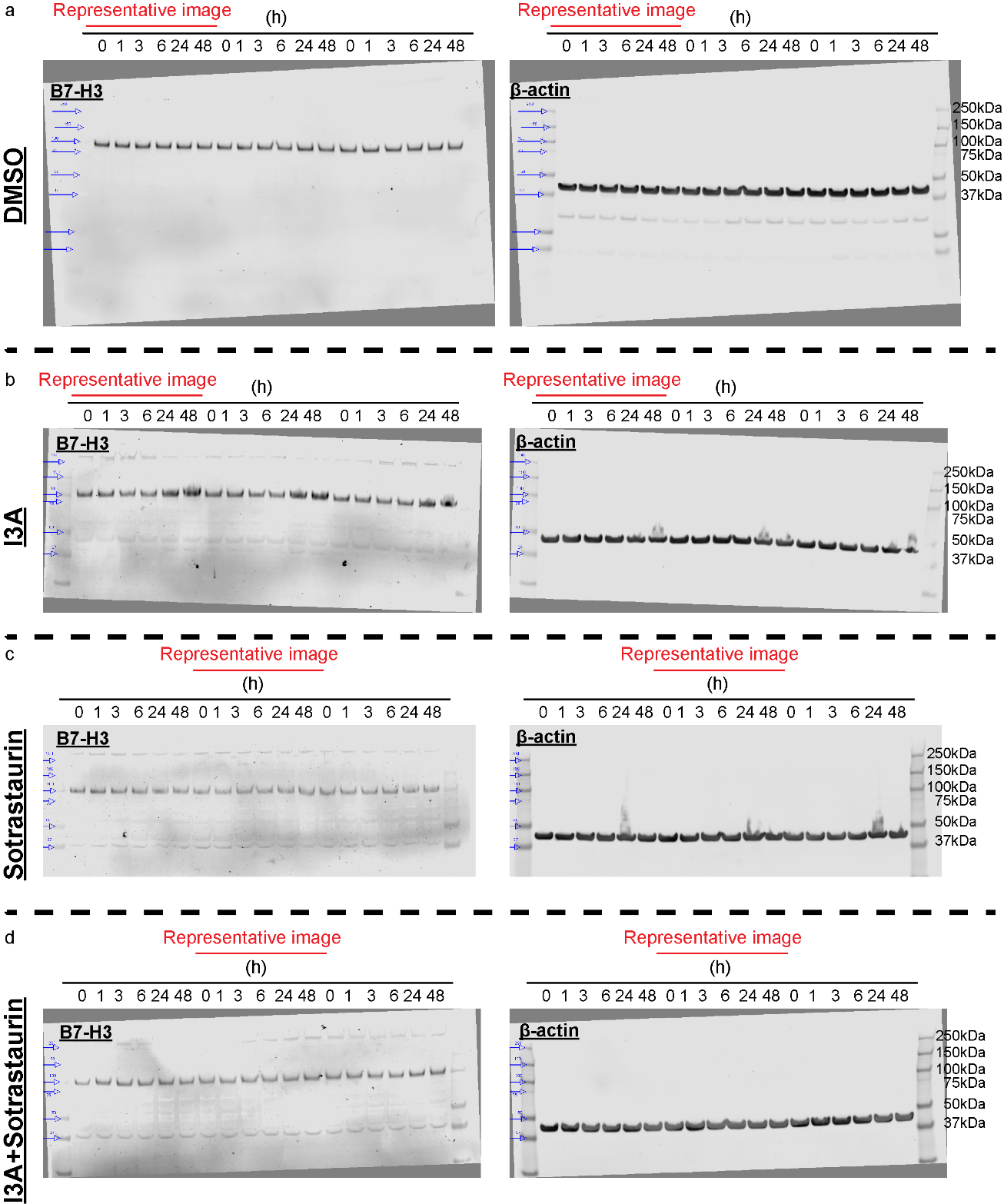


**Figure S8. Uncropped western blot images of representative images in Figure 3d.** Three biological triplicates are blotted on a single membrane. Representative blots shown in Figure 3d are highlighted by red lines over the lanes for **a** DMSO, **b** I3A only, **c** sotrastaurin only, or **d** I3A plus sotrastaurin. Molecular weight markers are shown in the leftmost and rightmost lanes and their molecular weights are labeled on the right.

**Figure S9.**


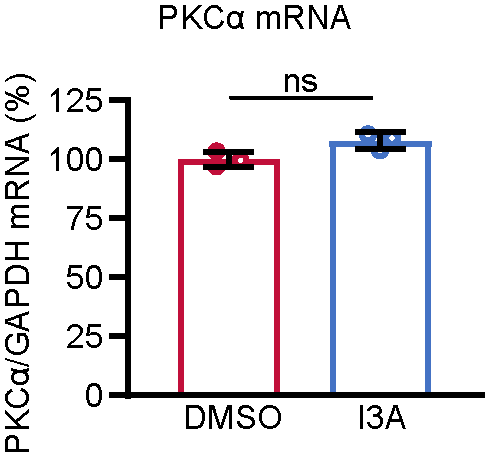


**Figure S9.** **I3A does not change the PKCα mRNA level in LM7 cells.** LM7 cells were treated with 0.5 µM I3A or DMSO for 48 hours (N=3). Total RNA was isolated from the cells and reverse-transcribed to cDNA. PKCα mRNA expression level was detected by RT-qPCR. Data represents mean ± SD. ns, non-significant by unpaired t-test.

**Figure S10.**


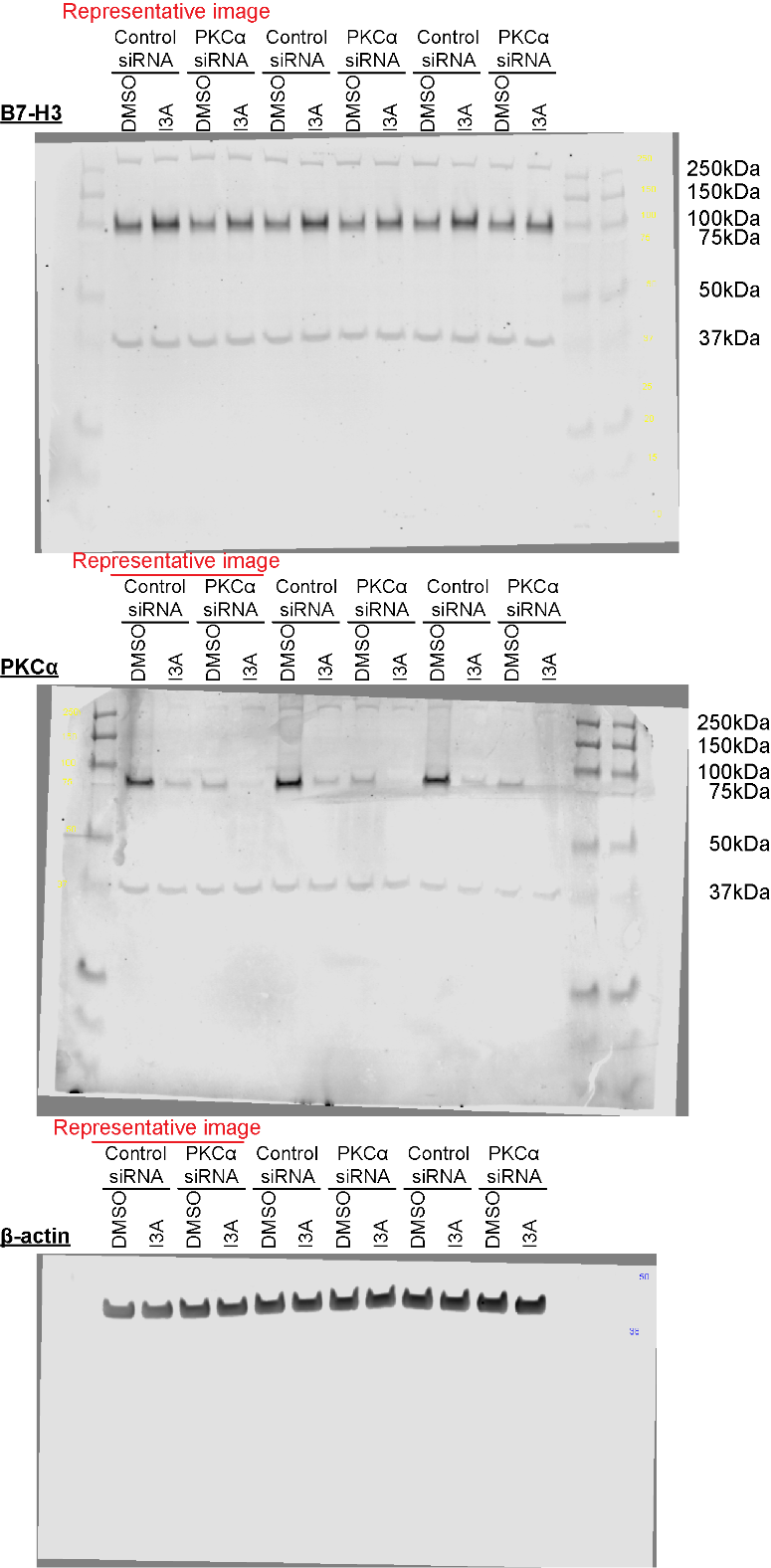


**Figure S10. Uncropped western blot images of representative images in Figure 4b.** Three biological triplicates are blotted on a single membrane. The representative blots shown in Figure 4b are highlighted by red lines over the lanes. Molecular weight markers are shown in the leftmost and rightmost lanes and molecular weights labeled on the right. Molecular markers on the membrane for beta actin blots are not shown because the intensity of the molecular markers is much lower than the intensity of beta actin blots in the image. However, when the maximum brightness is reduced, the molecular markers become visible (not shown), and the positions of the 50 kDa and 38 kDa markers are marked accordingly.

**Figure S11.**


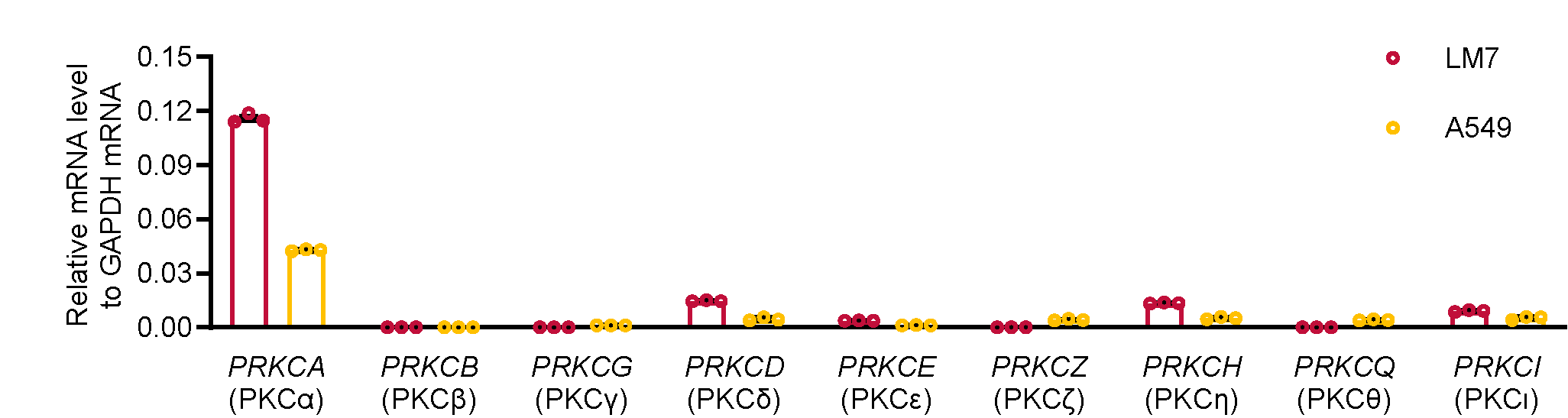


**Figure S11. mRNA levels of nine PKC isoforms in LM7 and A549 were measured by RT-qPCR.** PKC isoform mRNA levels were normalized by GAPDH mRNA levels (N=3). The result of LM7 cells in this graph is the reanalyzed result from Figure 4a. Data represents mean ± SD.

**Figure S12.**


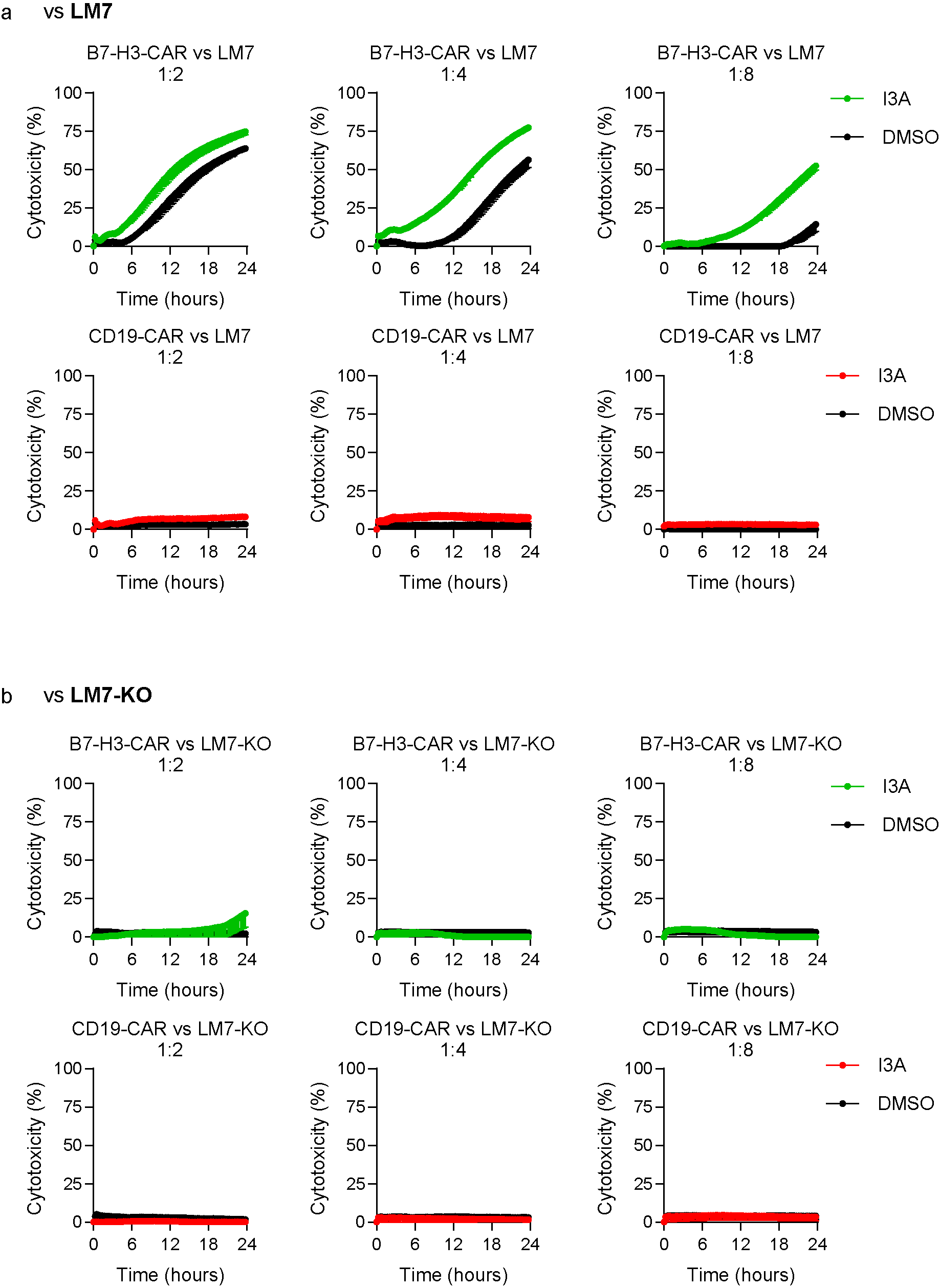


**Figure S12. I3A induced B7-H3 expression enhanced B7-H3-CAR T cell cytotoxicity.** B7-H3- or control (CD19)-CAR T cell cytotoxicity against **a** LM7 or **b** LM7-KO cells treated with DMSO or 0.1µM I3A (N=3). Data contains all time points (0 to 24 hours) for the experiments shown in Main Figure 5c. Data represents mean ± SEM (a and b).

**Figure S13.**


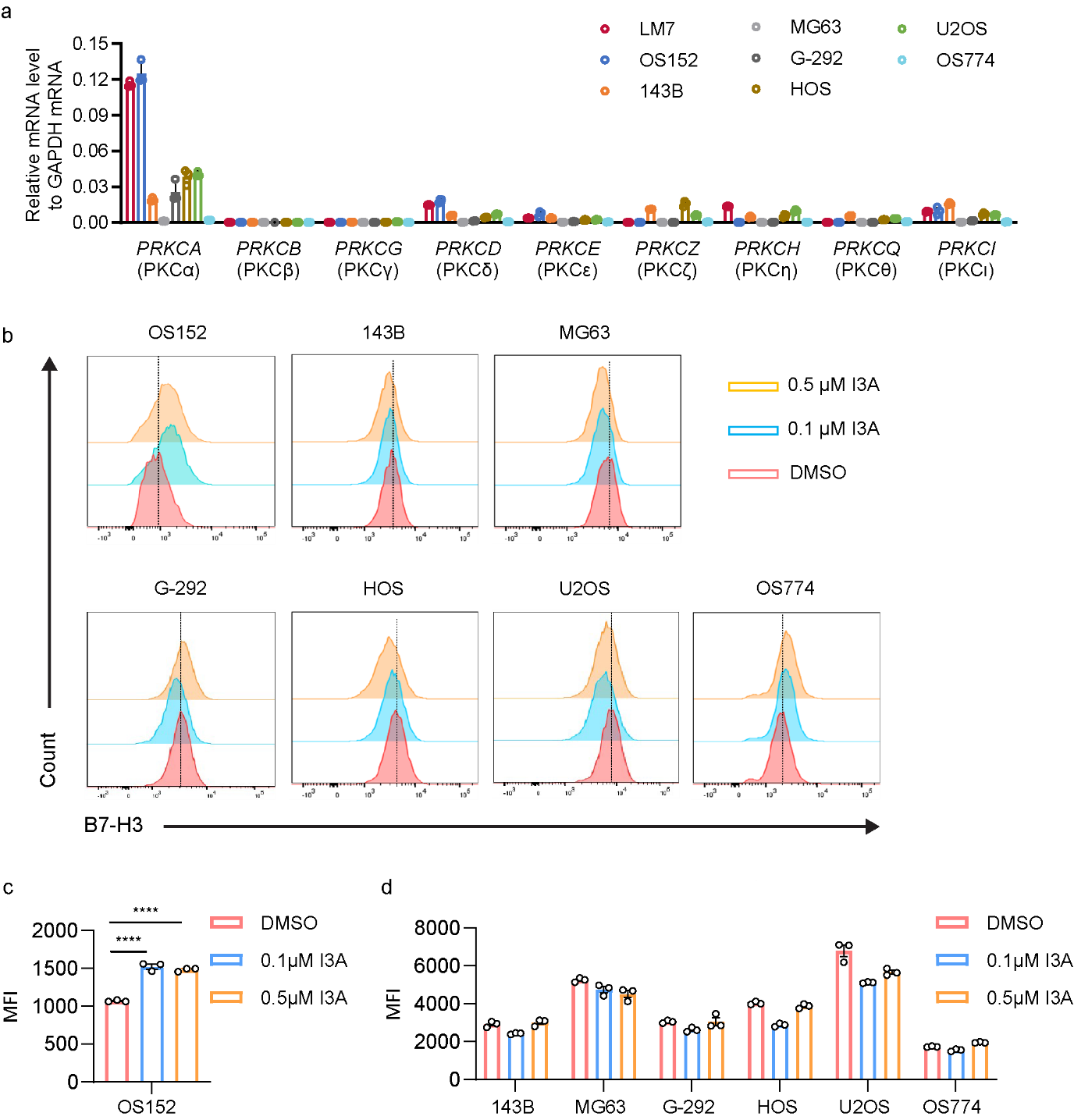


**Figure S13. I3A increased B7-H3 expression in PKCα high OS152 cells and not in PKCα low 143B, MG63, G-292, HOS, U2OS or OS774 cells. a** mRNA levels of nine PKC isoforms in osteosarcoma cell lines were measured by RT-qPCR. PKC isoform mRNA levels were normalized by GAPDH mRNA levels. The result of LM7 cells in this graph is the reanalyzed result from Figure 4a and the same data depicted in Figure S11. **b-d** Osteosarcoma cell lines were treated with 0.5uM DMSO, 0.1µM I3A, or 0.5µM I3A for 48 hours and B7-H3 expression was quantified by flow cytometry. **b** Representative flow plots for all cell lines. **c** Summary data for OS152 (N=3; technical replicates) and **d** cell lines with no increased B7-H3 expression (N=3; technical replicates). Data represents mean ± SD (a) or mean ± SEM (b, c, and d). ****p<0.0001 by one-way ANOVA (c). MFI, mean fluorescence intensity.

**Figure S14.**


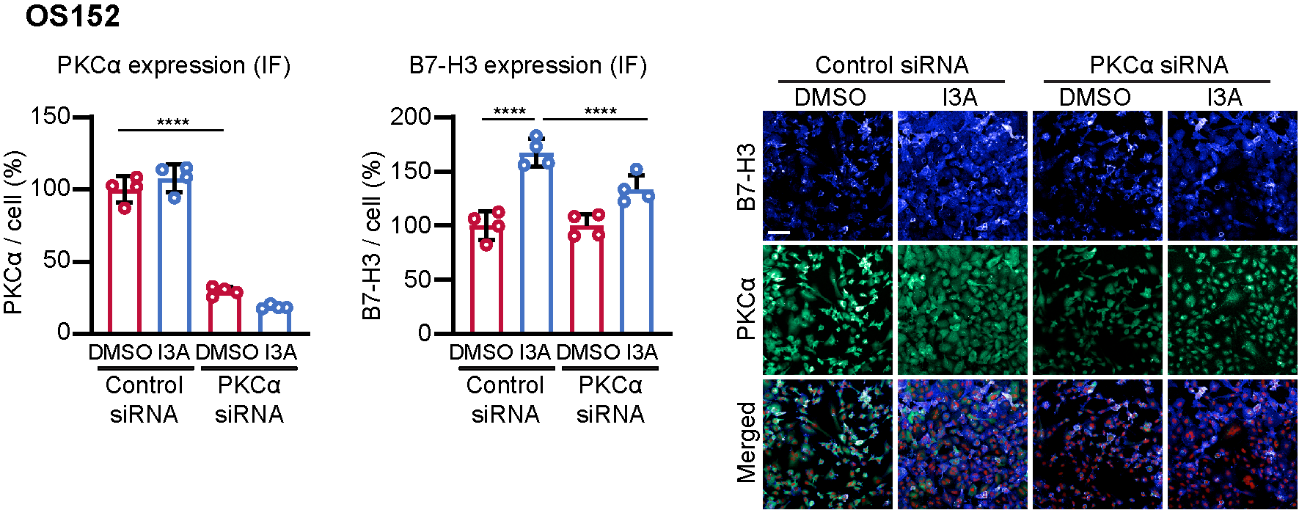


**Figure S14. PKCα is required for I3A induced B7-H3 protein expression in OS152 cells.** OS152 cells were knocked down using control or PKCα siRNA. After knockdown and treatment with DMSO or 0.5 µM I3A for 48 hours, PKCα (left panel) and B7-H3 (middle panel) protein levels were determined using the immunofluorescence assay (N=4). Representative immunofluorescence images are shown (right panel). B7-H3 (extended blue), PKCα (green), and nuclei (red). Scale bar = 100μm. Data represents mean ± SD. ****p<0.0001 by two-way ANOVA.

**Figure S15.**


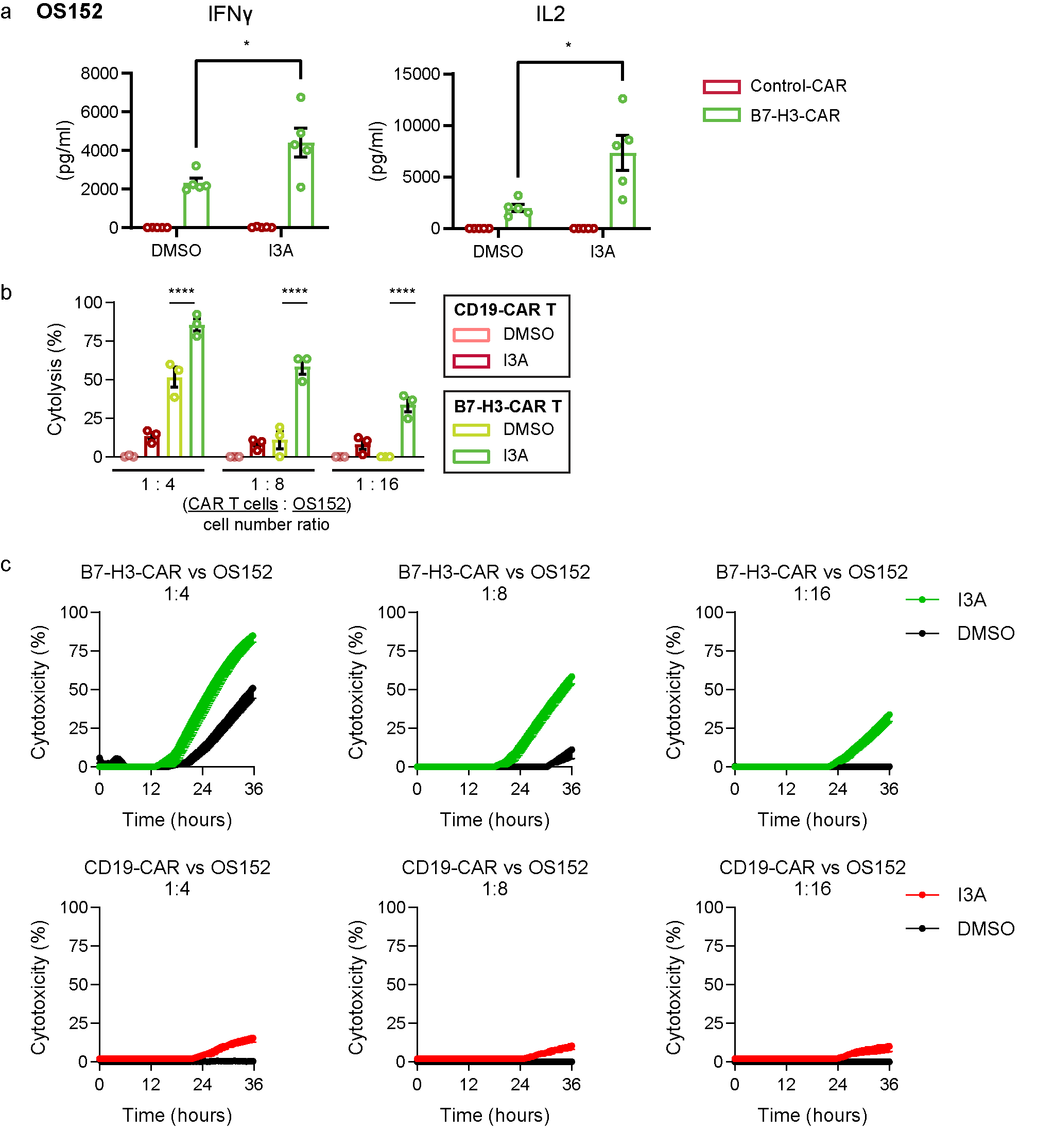


**Figure S15. I3A induced B7-H3 expression enhances B7-H3-CAR T cell effector function against OS152 osteosarcoma cells. a** Control (CD19)-CAR or B7-H3-CAR T cell IFNγ (left panel) and IL2 (right panel) secretion 24 hours post coculture with OS152 cells treated with DMSO or 0.1 µM I3A (N=5). **b,c** OS152 cells were treated with DMSO or 0.1µM I3A for 48 hours followed by B7-H3-CAR or CD19-CAR T cells. Cytotoxicity was quantified using an impedance-based assay. Cell index was monitored every 15 minutes and normalized prior to the addition of drug. **b** Summary data and **c** all time points post coculture (0 to 36 hours) are shown (N=3). Data represents mean ± SEM (a, b, and c). *p<0.05, ****p<0.0001 by two-way ANOVA (a and b).

**Table S1.**

| **Compound name** | **Mechanism** | **>50% increase** | | | |
| --- | --- | --- | --- | --- | --- |
|  |  | **Total B7-H3 signal/cell** | | **Mean B7-H3 signal/cell** | |
|  |  | **24 hours** | **48 hours** | **24 hours** | **48 hours** |
| ADEFOVIR DIPIVOXIL | Reverse-transcriptase inhibitor |  | Y |  |  |
| AFATINIB | ErbB family inhibitor |  |  |  | Y |
| ALLOPURINOL | Xanthine oxidase inhibitor |  | Y |  | Y |
| AURANOFIN | Redox enzyme inhibitor |  |  |  | Y |
| AXITINIB | VEGF receptor inhibitor |  | Y |  |  |
| BLEOMYCIN | Glycopeptide antibiotic |  | Y |  |  |
| BMY-40481-30 | Topoisomerase II inhibitor |  | Y |  |  |
| CAPECITABINE | Antimetabolic agent |  | Y |  |  |
| CARFILZOMIB | Proteasome inhibitor |  |  |  | Y |
| CEFPIRAMIDE ACID | Penicillin-binding protein |  | Y |  | Y |
| CICLOPIROX | Polyvalent cation chelator |  | Y |  |  |
| CLADRIBINE | DNA synthesis and repair inhibitor |  | Y |  |  |
| CORTISONE ACETATE | Glucocorticoid receptor binder |  | Y |  |  |
| CYCLOPHOSPHAMIDE | Alkylating agent |  | Y |  |  |
| CYTARABINE | Antimetabolic agent |  | Y |  |  |
| DACTINOMYCIN | Transcription inhibitor |  |  |  | Y |
| DASATINIB | Src kinase inhibitor |  |  | Y | Y |
| DAUNORUBICIN | Topoisomerase II inhibitor |  | Y |  | Y |
| DEFERASIROX | Iron chelator |  | Y |  |  |
| DEFEROXAMINE MESYLATE | Iron chelator |  | Y |  |  |
| DIPYRIDAMOLE | phosphodiesterase inhibitor | Y | Y | Y | Y |
| DOXORUBICIN | Topoisomerase II inhibitor |  |  |  | Y |
| FENOFIBRATE | PPAR alpha activator |  | Y |  | Y |
| FLOXURIDINE | DNA synthesis inhibition |  |  |  | Y |
| FLUDARABINE PHOSPHATE | DNA synthesis inhibition |  | Y |  |  |
| GEMCITABINE | DNA synthesis inhibition |  | Y |  |  |
| HOMOHARRINGTONINE | Protein synthesis inhibition |  |  |  | Y |
| IDOXURIDINE | Antiviral agent |  | Y |  | Y |
| INGENOL 3-ANGELATE | Protein kinase C activator |  | Y | Y | Y |
| IRINOTECAN | Topoisomerase I inhibitor |  | Y |  | Y |
| METHENAMINE | Antimicrobial activity |  | Y |  | Y |
| MITOXANTRONE | Topoisomerase II inhibitor | Y |  |  | Y |
| OXYPHENBUTAZONE | Cyclooxygenase inhibitor |  |  |  | Y |
| PONATINIB | Multi-tyrosine kinase inhibitor | Y |  | Y | Y |
| PROCARBAZINE | Alkylating agent |  | Y |  | Y |
| PYRITHIONE ZINC | Antifungal effect |  |  |  | Y |
| TENIPOSIDE | Topoisomerase II inhibition |  | Y |  | Y |
| TOPOTECAN | Topoisomerase I inhibitor |  |  |  | Y |
| TRIFLURIDINE | Viral replication inhibition |  | Y |  |  |
| TRIMETREXATE | Dihydrofolate reductase inhibition |  | Y |  |  |

**Table S1. Drugs that increased B7-H3 expression in the 5-point dose response assay.** Of the 55 drugs evaluated in the 5-point dose-response assay, 40 increased the total and/or mean B7-H3 signal per cell greater than 50% at one or more doses within 24 or 48 hours. The table includes each of these 40 drug names, mechanism of action, and the conditions tested. "Y" denotes conditions where a drug increased B7-H3 signal per cell by greater than 50%.
